# Release of Graphene and Carbon Nanotubes from Biodegradable Poly(Lactic Acid) Films during Degradation and Combustion: Risk Associated with the End-of-Life of Nanocomposite Food Packaging Materials

**DOI:** 10.3390/ma11122346

**Published:** 2018-11-22

**Authors:** Stanislav Kotsilkov, Evgeni Ivanov, Nikolay Kolev Vitanov

**Affiliations:** 1 Institute of Mechanics, Bulgarian Academy of Sciences, Acad. G. Bonchev, Block 4, 1113 Sofia, Bulgaria; ivanov_evgeni@yahoo.com (E.I.); vitanov@imbm.bas.bg (N.K.V.); 2 Research and Development of Nanomaterials and Nanotechnologies (NanoTechLab Ltd.), Acad. G. Bonchev, Block 4, 1113 Sofia, Bulgaria

**Keywords:** graphene, carbon nanotubes, poly(lactic) acid, degradation, combustion, fire, risk analysis

## Abstract

Nanoparticles of graphene and carbon nanotubes are attractive materials for the improvement of mechanical and barrier properties and for the functionality of biodegradable polymers for packaging applications. However, the increase of the manufacture and consumption increases the probability of exposure of humans and the environment to such nanomaterials; this brings up questions about the risks of nanomaterials, since they can be toxic. For a risk assessment, it is crucial to know whether airborne nanoparticles of graphene and carbon nanotubes can be released from nanocomposites into the environment at their end-life, or whether they remain embedded in the matrix. In this work, the release of graphene and carbon nanotubes from the poly(lactic) acid nanocomposite films were studied for the scenarios of: (i) biodegradation of the matrix polymer at the disposal of wastes; and (ii) combustion and fire of nanocomposite wastes. Thermogravimetric analysis in air atmosphere, transmission electron microscopy (TEM), atomic force microscopy (AFM) and scanning electron microscope (SEM) were used to verify the release of nanoparticles from nanocomposite films. The three factors model was applied for the quantitative and qualitative risk assessment of the release of graphene and carbon nanotubes from nanocomposite wastes for these scenarios. Safety concern is discussed in respect to the existing regulations for nanowaste stream.

## 1. Introduction

Manufactured nanomaterials are applied in various consumer goods in order to enhance their properties or to supplement novel functionalities. The industry has already utilized nanoclays, metal nanoparticles and carbon nanotubes and has managed to use them in products for a variety of applications, e.g., semiconductors, automotive, aerospace, electronics, energy, defense, sporting goods, and packaging [1]. Nanotechnology allows scientists to alter the structure of packaged materials on a molecular scale in order to give the materials the desired properties [2,3]. Nowadays, different types of carbon nanotubes and graphene in polymer nanocomposites are widely investigated for the development of smart, active and intelligent packaging that can improve the quality and safety of food, to solve the food storage problem and to inform the consumer about the quality of packaged food [2,3,4]. Graphene and multiwall carbon nanotubes (MWCNTs) in biodegradable polymers are the most continuous and potentially valuable nanoscale materials to have emerged in recent years, and are increasingly studied to enhance the thermal, mechanical, barrier properties and functionality of food packaging materials [1,2,3,5]. Among the many different biopolymers available, polylactic acid (PLA) is a promising alternative, especially for food packaging and biomedical packaging applications. However, a key challenge is to enhance the barrier properties of the PLA packaging. Graphene and its derivatives are identified as powerful candidates for gas-barrier materials because perfect graphene does not allow the diffusion of small gases through its plane [6,7,8]. Incorporation of graphene and carbon nanotubes into polymer matrices are also a promising nanotechnology approach to increase mechanical strength and improve thermal properties when properly dispersed in a polymer matrix [9,10].

At the same time, regulators at national, European and international level are still struggling to agree upon a unified and mutually accepted definition of a “nanomaterial”. As the market of nanomaterial-based products is expected to triple by the year 2020 in more industrial sectors, the application of nanomaterials and nanoparticles is expected to grow proportionally [2,3,11]. As a result, environmental exposure to nanoparticles in air, water and soils is also expected to increase [11]. Therefore, more research efforts in nanoscience are needed to focus exclusively on the potential risk of nanomaterials graphene and carbon nanotubes with the increasing exposition of consumers and the environment to nanoparticle-containing packaging. The specific nanoscale size and shape of graphene and carbon nanotubes with a large aspect ratio and large surface area, airborne, non-soluble in water and absorptive in soil, will enhance the risk of their mobility in the environment [12,13].

Currently, very little is known about the release of the nanomaterials graphene and carbon nanotubes (CNT) incorporated into polymer nanocomposites. Although they are typically tightly bound in the matrix polymer, their release through the lifecycle of nanocomposites is possible [11]. Therefore, greater information is needed on the potential hazard associated with specific exposure scenarios. Few scenarios were identified and published in the literature [11,14,15] where CNTs might be released into the environment during the life cycle of polymer nanocomposites in the production, service life and disposal stages. Release during service the life of CNT-based composites is projected to be quite low and composed of polydisperse fragments with only a small fraction of free single nanotubes [15,16,17]. The general conclusion is that the CNTs form a network and are not easily detachable from the samples, but the CNT layer on the surface of the degraded composites could be a source of a high quantity of released free standing CNTs and thus, may pose a health risk [16,17]. Researchers [16,17] reviewed the potential release of CNTs into the environment during the service life where untrained humans are in contact with oil-based polymer nanocomposites. They considered three possible pathways for the release of CNTs: due to exploitation and use, degradation of the matrix due to weathering processes, and fire events. Duncan and Pillai [18] assumed the nanoparticle release paradigms to be: (i) via passive diffusion, desorption and dissolution into external liquid media; and (ii) by matrix degradation. However, currently there are no standard methods to measure what is released from use of products containing nanomaterials.

Studies on the release of graphene through the lifecycle of nanocomposites are very scare. Arvidsson, et al. [12] reported that the potential environmental and health risk of graphene at the production stage could be great, depending on the type of synthesis methods [12]. For most materials, degradation of the polymer matrix during the service life is associated with the greatest potential for release, with rates dependent on the specific characteristics of the polymer, carbon nanofiller, and environment [15,16]. In our previous studies [19,20,21], we have reported on the release of graphene nanoplatelets from food packaging materials into food simulants, due to the migration processes and the partial degradation of the biodegradable polymer matrix. Therefore, the released graphene nanoplatelets and their aggregates from the nanocomposite wastes during their degradation may pose a health risk [16,17]. The fate of graphene in polymer nanocomposite exposed to UV radiation was discussed and researchers concluded that graphene nanoparticles would be able to pass into the air or engage in the soil and groundwaters [22].

End-of-life aspects of nanomaterials have received far less attention than their preparation or application [23]. The two main strategies used for the end-of-life of thermoplastics products are recycling and burning to produce energy (thermal valorization). As the release of nanoparticles during grinding of nanocomposite wastes presents a potential risk, the incineration of nanocomposites has recently been accepted as a prospective waste management strategy, for which nanoparticle emission during burning must be addressed as a premise. So far, no detailed study has been published that investigates the release of graphene from nanocomposites due to accident fire or burning. Few publications have discussed the incineration and burning of CNT-based nanocomposites [24,25]. In contrast to incineration, where under high temperatures CNTs can be destroyed [24], a fire or burning of CNT nanocomposites in open air may not degrade all CNTs particles in composites, since the decomposition temperature of CNTs is much higher than that of the polymer matrix. A network of CNTs is formed in the char (residue ash), but measurements are still missing if CNTs can be released from the residue ash into the air. Bouillard, et al. [25] reported on the release of free CNTs and agglomerates of CNTs from acrylonitrile–butadiene–styrene (ABS) nanocomposites into the air during nanowastes combustion at quite low temperatures (about 400 °C). This information was found to be important to assess the environmental risks and the inhalation risks to people engaged in those practices.

In the present study, we investigate the release of graphene nanoplates and multi-walled carbon nanotubes from biodegradable poly(lactic) acid nanocomposite films at various temperatures, due to polymer degradation and burning. We analyze the risk from the release of graphene nanoplatelets and multi-walled carbon nanotubes at the end-of-life of the nanocomposite wastes, due to biodegradation at composting, combustion and accident fire. The thermogravimetric analysis (TGA), transmission electron microscopy (TEM), atomic force microscopy (AFM) and scanning electron microscope (SEM) were used to verify the release of nanoparticles from nanocomposite films. The three-factors model (C.E.L.) was applied for the quantitative and qualitative risk assessment of the release of graphene and carbon nanotubes from nanocomposite wastes for these scenarios’ materials. Safety concerns are discussed in respect to the application of nanomaterials in food packaging, bio-medical packaging and others.

## 2. Materials and Methods

Nanocomposite of poly(lactic) acid polymer (PLA) filled with graphene nanoplatelets (GNP) and multiwall carbon nanotubes (MWCNTs) was supplied from Graphene 3D Lab, (Ronkonkoma, NY, USA) in the form of filament for 3D printing (FDM). Commercial neat PLA filament was also supplied. The matrix polylactic acid is CAS# 26100-51-6, Sigma-Aldrich, (St. Louis, MO, USA) with Mw~60,000 and 1.8% crystallinity. Disk samples having sandwich structure of 10 alternative layers of the nanocomposite and the PLA were 3D prepared with the dual extruder X400 Rep Rap printer (Feldkirchen, Germany). The printed samples were hot pressed to thin films with a thickness of 30 microns. The amount of nanocarbon filler in the final film samples is 3 wt. %.

The film samples were emerged in two aqueous-based solvents of 10 vol. % ethanol and 50 vol. % ethanol. Ultra-strong migration test was performed with heating at 90 °C for 4 h, followed by storage for 10 days at 40 °C and dynamic treatment for 1 min daily. The migration test regime was applied to simulate the conditions of high temperature treatment of nanocomposite packaging during their end-of-life as waste disposal.

Different visualization techniques were applied in order to identify migrants in the simulant media and to verify the film integrity after the migration test. TEM (EOL JEM 2100, Preabody, MA, USA) at accelerating voltage 200 kV and AFM (Bruker, Billerica, MA, USA) were used for the analysis of the dried colloids of migrated substances into the surrounding solvents. For preparation of the test samples, a micro-quantity of colloid after migration test was dropped on a copper TEM grid covered by a membrane from amorphous carbon (or on glass plate for AFM scan), and after that dried in a dust-free atmosphere at ambient conditions. The morphology of the film surface before and after the migration test was studied by scanning electron microscope (Philips 515, Eindhoven, Netherlands) with accelerating voltage 25 kV and 5 kV. Before the examination in the microscope, the samples were covered with metal coating for better conductivity of the surface and to avoid discharge effects.

Thermal stability and degradation of nanocomposite films were studied by TGA (Q50, TA Instruments, New Castle, DE, USA), in air atmosphere, at a heating rate of 10 °C/min in three different temperature ranges, from 30 °C to 500, 650 and 850 °C. The mass loss during heating and the amount of residue ash were analyzed. TEM was performed for analysis of the residue ash after burning at the three temperatures.

Risk assessment analysis was performed using the three factors method, 3F or C.E.L., i.e., grading the three risk analysis factors: Consequences (C), Exposure (E), and Probability/Likelihood (L).

## 3. Results and Discussions

### 3.1. Release of Graphene and Carbon Nanotubes Due to Degradation of PLA Polymer

The use of biodegradable packaging materials, such as PLA will contribute to sustainability and reduction of wastes via degradation [26,27]. Composting, for example, has the potential to transfer biodegradable waste, including biodegradable plastics, into useful soil amendment products by an accelerated degradation using a mixed microbial population in a moist, warm, aerobic environment under controlled conditions. Song, et al [26] found that biodegradable packaging materials are most suitable for single-use disposable applications where the post-consumer waste can be locally composted. However, special care should then be taken while handling local composting of biodegradable nanowastes to limit potential environmental risks due to the release of nanoparticles in the soil from the compost.

We discuss herewith if the GNP and MWCNTs release as single nanoparticles or large aggregates from the nanocomposite packaging films via degradation of the PLA matrix. The characterization of such release provides critical information for environmental nano-object exposure from biodegradable nanocomposites. In our previous studies [19,20,21], we have investigated the release of GNP and MWCNTs from the composite film GNP/MWCNT/PLA in alcoholic and acid food simulants, at high temperature migration conditions, such as: (i) strong static migration test (heating at 90 °C for 4 h); and (ii) ultra-strong dynamic migration test (heating at 90 °C for 4 h followed by subsequent storage for 10 days at 40 °C, including dynamic treatment for 1 min daily). The migration conditions were set accordingly with the prescription in EU Regulation 10/2011 (EU 2011) [28] and literature sources [29,30].

We have observed that large graphene nanoplatelets (GNP) of about 100–1000 nm in length and a few nanometers in thickness indeed migrate from the GNP/MWCNT/PLA film into the food simulants, due to the diffusion processes [19,20]. During the strong static migration test, the total amount of released substances (nanoparticles and organic matter) from the composite GNP/MWCNT/PLA films was estimated around 0.028–0.053 mg/cm^2^, where the nanoparticle migrants are of 0.006–0.011 mg/cm^2^, depending on the food simulants [20]. Therefore, the released substances after this test remain below the overall migration limit (OEL = 0.10 mg/cm^2^) for food contact material accepted by the EU regulatory documents [28,31].

By contrast, during the ultra-strong dynamic migration test [20], the release of nanoscale size particles (100–1000 nm) from the GNP/MWCNT/PLA composite film is higher (0.5–0.7 number %) compared to (0.1–0.2 number %) nanoparticle migrants that were observed during the strong static migration test in the three food simulants, 3% acidic acid, and 10% and 50% ethanol. The larger size nanoparticle migrants (1–10 microns) were found also in higher amounts (3–5 number %) during the ultra-strong migration test, compared to 1–2 number % for the strong static test. Laser diffraction analysis was used in our previous studies [19,20] to detect the number and size distribution of the released nanoparticles from GNP/MWCNT/PLA films into the food simulants. In general, a threefold higher number of nanoparticles are observed to release during the ultra-strong dynamic migration test (heating at 90 °C for 4 h followed by storage for 10 days at 40 °C and dynamic treatment for 1 min daily) compared to the strong static test above. The detected nanoscale particles (100–1000 nm) in the food simulants are 0.5–0.7% while the micron scale agglomerates (1–10 µm) are 3–5%, compared to 0.1–0.2% nanoparticles and 1–2% agglomerates released after the strong static conditions [20]. This was associated with partial degradation of the PLA polymer matrix, which supports the diffusion of GNPs together with dissolved organic substances out of the film. However, the MWCNTs form the entangled network in the polymer film, which prevents their migration into food simulants if the polymer partially degrades.

**Figure 1:**
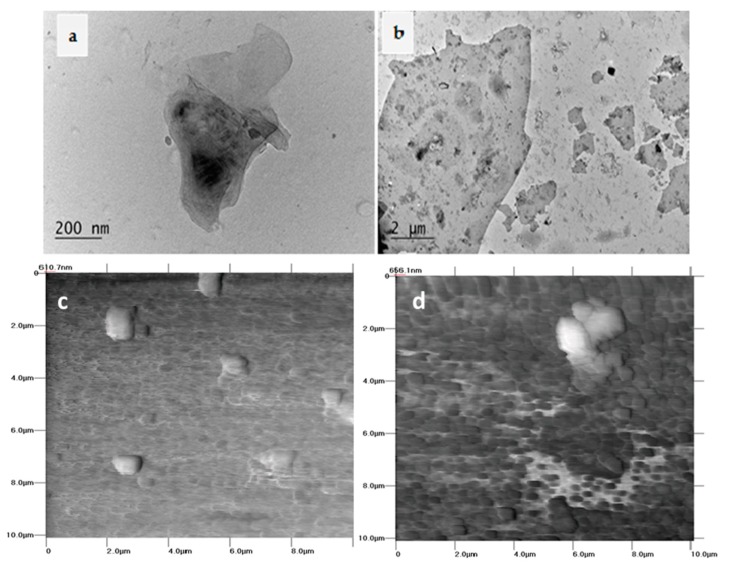
(**a**,**b**) transmission electron microscopy (TEM) micrographs and (**c**,**d**) atomic force microscopy (AFM) scan of dried migrants from GNP/MWCNT/PLA film after the ultra-strong dynamic test in 10% ethanol (first column) and 50% ethanol (second column).

**Figure 2:**
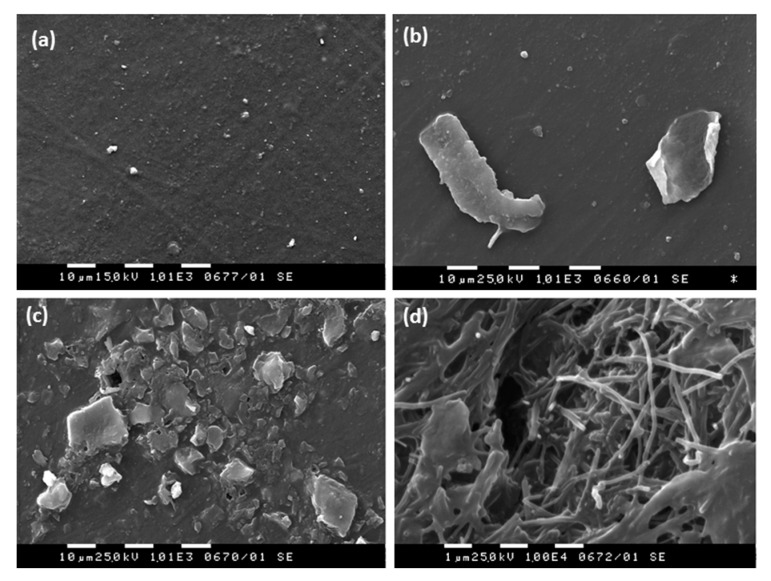
Scanning electron microscope (SEM) micrographs of the film surfaces: (**a**) reference GNP/MWCNT/PLA film before the test; (**b**) after the ultra-strong dynamic test in 10% ethanol, (**c**,**d**) after the ultra-strong dynamic test in 50% ethanol (in different places).

Based on upper results, we discuss herein the scenario of nanoparticle release due to degradation of GNP/MWCNT/PLA composite films at the end-of-life stage of disposal or composting the wastes. Figure 1 presents the TEM micrographs (Figure 1a,b) and AFM scans (Figure 1c,d) of the dried migrants in 10% and 50% ethanol after an ultra-strong dynamic migration test. In Figure 2, the SEM micrographs of the film surfaces before and after such treatment are compared.

As seen from the TEM micrographs in Figure 1a,b, mainly small aggregates of GNPs below 500 nm are observed in a low amount in 10% ethanol (Figure 1a). While in a more aggressive media of 50% ethanol (Figure 1b), the amount of released GNPs increases apparently, and most of the platelets are in nanoscale size, 100–1000 nm; only a few aggregates of size 1–10 µm are visible. The AFM scans of dried colloids in 10% ethanol (Figure 1c) visualize the presence of a small amount of particles in a size of about 1 µm. However, in 50% ethanol (Figure 1d), the migrants are many small objects with a nanoscale size below 500 nm and a large aggregate of ~2 µm length and thickness of ~500 nm. Importantly, MWCNTs are not visible to release from the GNP/MWCNT/PLA nanocomposite films, as observed by TEM and AFM analysis. Following the release mechanisms of nanoparticles proposed by Duncan and Pillai [18], we assume that the physical changes of biodegradable PLA polymer due to polymer hydrolysis provokes the diffusion of the graphene nanoplatelets and the dissolved organic substances out of the film into the food simulant.

SEM analysis was performed in order to examine the GNP/MWCNT/PLA film integrity before and after the ultra-strong dynamic migration test in the two alcohol-based simulants. The film surface before the treatment (Figure 2a) is smooth and free from ingredients. For the film immersed in 10% ethanol (Figure 2b), a few graphene nanoplates of a size of above 10 µm are extracted on the film surface. By contrast, in 50% ethanol (Figure 2c,d), the integrity of the GNP/MWCNT/PLA composite film is destroyed due to the partial degradation of the PLA polymer and much extraction of substances on the film surface. It is visible that the fibrous MWCNTs formed an entangled network as the PLA polymer matrix dissolved, which prevented their release into the food simulant.

It may be concluded based on the above results, that if the biodegradable nanocomposite wastes containing GNPs and MWCNTs are disposed in landfills, nanoscale graphene platelets can certainly be released into the environment due to partial degradation and weathering. If such nanowastes are subjected to composting, the biodegradation process provides compost that is very likely to contain large amounts of nanoscale GNPs, as well as graphene aggregates and bundles of carbon nanotubes of micron size. Since the compost is intended to be used for soil improvement, those nanoparticles would penetrate into soil and groundwater, and there is a potential risk of falling into the food chain of different organisms.

### 3.2. Release of Graphene and Carbon Nanotubes Due to Burning of Nanowastes

Burning of nanocomposite wastes to produce energy (thermal valorization) has been recently discussed as a nanowaste management strategy, and thus the risks for nanoparticle emission during incineration of thermoplastic nanocomposites must be addressed and investigated [25]. Moreover, the treatment of such waste by accidental fire or burning in landfills (a common practice in underdeveloped regions) may pose questions associated with environmental and human risks due to the potential release of large amounts of nanoparticles into the environment. In principle, graphene and CNTs are combustible materials above 600 °C, and they can be easily transformed into CO/CO_2_ during degradation [25]. The published results on this subject are very scarce, but a few papers [32,33,34] have reported that the combustion of polymer composites with CNTs could form residues (ashes) containing unburned CNTs. Moreover, the CNTs also may release in the combustion gas phase [25]. Therefore, in the present work, we classify burning of nanowastes as a scenario that may have a greater possibility to release airborne nanoparticles. The characterization of such a release may provide critical information for environmental and human accidental nanoparticle exposures.

**Figure 3:**
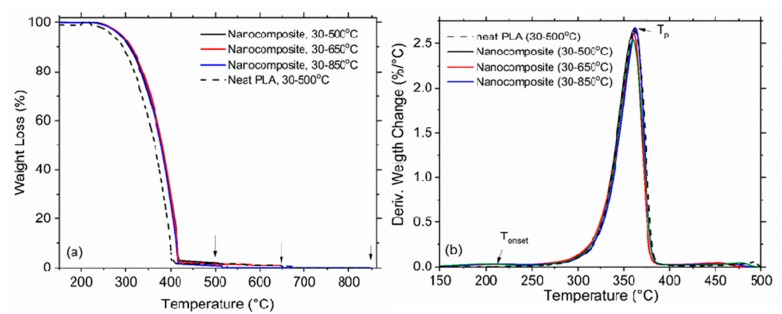
TGA (**a**) and DTG (**b**) curves of the neat PLA and GNP/MWCNT/PLA nanocomposite film compared to the neat PLA at three temperature regimes, 500, 650 and 850 °C, in an air atmosphere.

TGA was performed to simulate the nanowaste combustion in three heating regimes: 30–500 °C, 30–650 °C and 30–850 °C, at a heating rate 10 °C/min in air atmosphere. The graphene nanoplatelets used in this study are one of the possible forms of graphene-related materials. In general, the obtained thermal stability and the release results will depend strongly on the particular graphene material used in the nanocomposite. The thermal decomposition of the neat PLA and the nanocomposite films (GNP/MWCNT/PLA) were analyzed by the thermal gravity analysis (TG) and Differential thermal gravity (DTG) curves, which present the weight loss (%) and its first derivative versus temperature (°C), as shown in Figure 3a,b, respectively. The initial decomposition temperature (T_onset_), the decomposition peak temperature (T_p_) and the residue ash (%) were evaluated from the TG/DTG curves and data are presented in Table 1. The T_onset_ of the nanocomposite is observed around 230 °C, while T_p_ appears at 361 °C, which are higher than 10 °C and 5 °C, respectively, compared to the neat PLA. This indicates that the thermal stability of PLA is improved by the addition of 3 wt. % mixed nanofillers, GNP and MWCNT. As might be expected, the weight loss increases with increasing the heating temperature (T_max_) from 500 to 850 °C. The combustion of the neat PLA at 500 °C results in 0.3% residue from the initial weigh of the polymer sample and the ash consists of amorphous carbon (CB). In contrast, the combustion of GNP/MWCNT/PLA forms residue ash of 3.3% at 500 °C, 1.5% at 650 °C and 0.07% at 850 °C, containing mostly unburned nanoparticles, GNP and MWCNT. Therefore, further potential environmental problems may arise with handling such residue ash. Our study advances the observations in References [25,32,33,34] by showing that the amount of GNPs and MWCNTs in the residue ash decreases by increasing the burning temperature, this indicating increased decomposition of carbon nanoparticles by controlled incineration/combustion temperatures.

**Figure 4:**
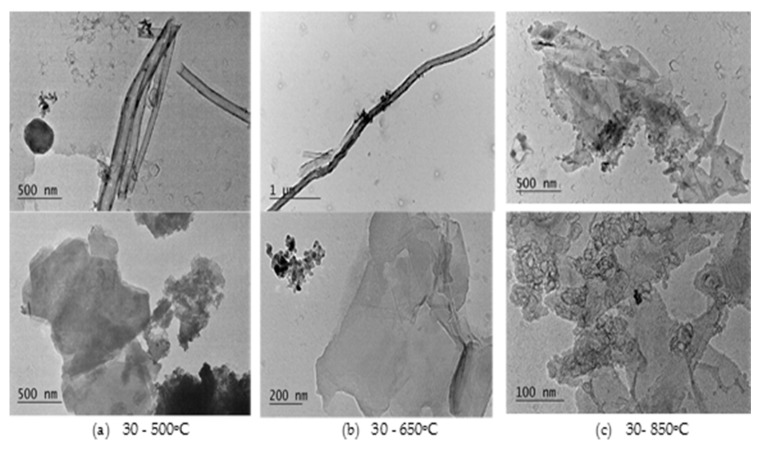
TEM micrographs of MWCNTs (first line) and GNPs (second line) observed in the residue ash at the three decomposition temperatures: (**a**) 500 °C, (**b**) 650 °C and (**c**) 850 °C in air atmosphere.

TEM micrographs in Figure 4 visualize the content of the residue ash after combustion at the three temperatures in air atmosphere. As seen in the first column (Figure 4a), at 500 °C, the residue ash (3.3%) is completely composed of unburned single MWCNTs and GNPs, or their loose agglomerates. At 650 °C (Figure 4b), the residue decreases to 1.5% and consists mostly of single airborne particles, MWCNTs and GNP, and some soot CB nanoparticles of primary sizes of 10–30 nm. The nanotubes are about 30 nm in diameter and a few microns in length and are very similar to the original MWCNT size. Similar findings are observed for the GNP particles. While at 850 °C (Figure 4c), the amount of the residue ash strongly decreases to 0.07%, confirming that the carbonaceous fillers are mostly degraded. Indeed, the MWCNTs are missing in the residue ash, but unexpected content of GNP particles is observed and they are mainly displayed as fractal aggregates mixed with some soot nanoparticles.

The observations in Figure 4 reveal that large amounts of single isolated airborne MWCNTs (<50 nm diameter and >1 µm length) and GNPs (>100 nm), as well as their loose fractals (1–2 µm) can be released during burning in air atmosphere, addressing, therefore, a new kind of safety issue with regards to the combustion/incineration of nanowastes or accidental fires. The airborne particles of GNPs and MWCNTs may either stay in the char residues, or may be released in the gas phase during incineration or fire. Their fate depends on the local operating conditions of the burning process.

### 3.3. Risk Assessment Associated with End-of Life of Nanocomposite Food Packaging Materials

Risk could be defined as a combination of the probability of occurrence of an event and its consequences, establishing a negative outcome. The methodology we applied to analyze and quantify the risk is borrowed from the standards and guidelines presented in several regulatory documents, such as: Risk Management Standard ISO 31000:2009 [35], British Standard BSI 2007 on safe handling and disposal of manufactured nanomaterials [36], USEPA Guidelines for Ecological Risk Assessment [37], and the British CSIRO Safe Handling and Use of Carbon Nanotubes [38]. In this study, the risk is mainly defined according to standard ISO 31000:2009 [35] as a comprehensive process of analysis and categorization, where the risk could be assessed quantitatively or qualitatively, depending on the probability of occurrence of the possible consequences.

To quantify the risk (R) of the release of MWCNTs and GNP from 3 wt. % GNP/MWCNT/PLA film at the end-of-life, as food packaging wastes, we have adopted the three factors method (C.E.L.), i.e., grading the three risk analysis factors: Consequences (C), Exposure (E), and Probability/Likelihood (L). The C.E.L. model is a widely recognized method of analysis and quantitative risk assessment [39,40]. Therefore, we have applied it for risk assessment in the four most popular scenarios for treatment of the food packaging wastes: biodegradation, combustion, burning in open air and accidental fire. The risk analysis factors are defined according to the C.E.L. model [39].

Consequences (C) represent the undesired results of an event or series of events. In this work, consequences are determined from the amount of the released MWCNTs and GNPs, as graded according to the recommended exposure limit of Carbon Nanotubes and Nanofibers (μg) in air (1 m^3^), REL = 1 μg/m^3^, proposed by NIOSH [41]. The NIOSH REL is expected to reduce the risk for pulmonary inflammation and fibrosis. Thus, six grades from 1 to 100 [39] are used for the quantification of consequences (xREL = 1 to > 1,000,000), as shown in Table 2.

Exposure (E) shows how often a certain danger can occur and how much the system is often threatened by accidents. The exposure estimates are based on the E-classification method [39], with six steps in the range from 0.5 to 1 (Table 2).

Likelihood (L) shows how likely it is to have consequences. The following six steps of grades from 0.5–10 are used to quantify this factor, as shown in Table 2.

The risk (R) is defined as the quantity comprised of the product of the three parameters: consequences (C), exposure (E) and probability (L): R = C × E × L. The eligibility of risk to health and environment is classified in the following five risk areas presented in Table 2, namely: minimal, acceptable, high, very high and unacceptable (hazard), depending on the calculated values of risk (R) varying from <20 to >400 [39]. The end-results of the risk assessment determine the eligibility of the identified risk and the need to apply measures to prevent or limit it.

#### Quantitative and Qualitative Risk Assessment for the Release of GNP and MWCNTs

For the quantitative risk assessment, we used the data obtained above for the release of GNP and MWCNTs from 3 wt. % GNP/MWCNT/PLA film, during the degradation in ultra-strong dynamic test, as well as the burning at three heating temperatures 500, 650 and 850 °C in air atmosphere. Importantly, for the biodegradation (i.e., composting, weathering), we assumed that the total amount of 3 wt. % GNPs/MWCNTs nanofiler may release from the GNP/MWCNT/PLA nanocomposite film in the form of agglomerates and single nanoparticles due to the full degradation (hydrolysis) of the PLA polymer. While for the burning processes like: combustion, burning in open air and accidental fire, the released GNPs and MWCNTs will depend on the heating temperature. The results from thermogravimetric analysis in Table 1 are used to simulate the combustion at the three temperatures in air atmosphere. For analysis of risk to humans and the environment, the released GNPs and MWCNTs are estimated for 100 kg wastes. Moreover, we have assumed that the single airborne nanoparticles of GNPs and MWCNTs (≤100 nm) and CB nanoparticles, that may release in 1 m^3^ air or soil are only 1% of the total amount of released nanoparticle agglomerates during the four scenarios studied.

The admissibility of the human and environmental risk is presented as a multiplication of the three factors (R = C × E × L). Table 3 presents the three risk analysis factors and the quantitative risk assessment for the four scenarios: biodegradation; combustion at 500, 650 and 850 °C; burning in open air (at 500 °C); and accidental fire (at 500 °C). As seen, the risk of GNPs and MWCNTs from the biodegradation is R = 270, while the risk from combustion varies within R = 100–1500 depending on the heating temperature (500–850 °C). However, the risk from the burning of wastes in open air (in landfills) is quite high (R = 180), while that from accident fire is low of R = 22.5, due to the very rare exposure.

For qualitative risk assessment, we used the approach described by Aven [40], which represents the dependence of the “consequences” in a function on the “frequency” of occurrence, the last being a derivative of “exposure” and “likelihood”. This dependence is shown in Figure 4, for the purpose of classification of the risk of MWCNTs and GNP release from 100 kg of 3 wt. % PLA/MWCNT/GNP nanocomposite wastes, under the scenarios of biodegradation, combustion (500–850 °C), burning in open air (at 500 °C) and accidental fire (at 500 °C).

The lines represent the “constant risk” at the following levels of risk: R = 20, R = 70, R = 200 and R = 400, and outline the five risk zones: minimum (R < 20); acceptable (20 < R < 70); high (70 < R < 200); very high (200 < R < 400) and hazard (R > 400). The experimental points represent the risk of exposure to MWCNTs and GNPs from the PLA nanocomposite film throughout the four scenarios of the waste treatment, listed in Table 3.

**Figure 5:**
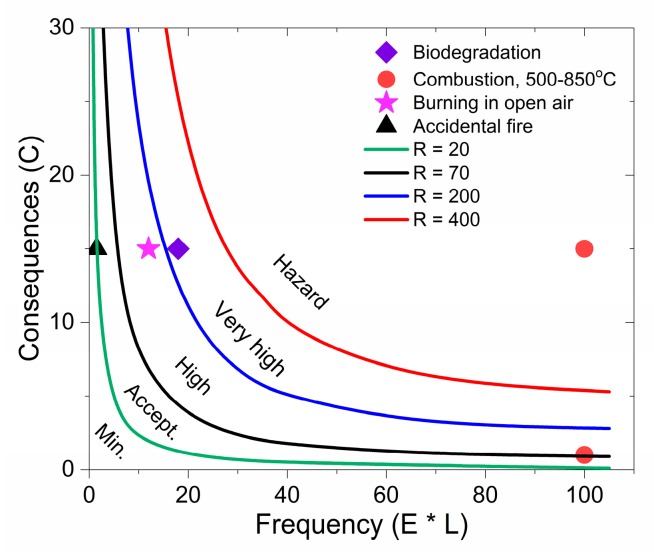
Qualitative risk assessment: consequences vs. frequency of occurrence of GNP and MWCNT exposure, related to four scenarios at the end-of life of 3 wt. % GNP/MWCNT/PLA packaging films: biodegradation, combustion, burning and accident fire. Risk is estimated for 100 kg nanocomposite wastes.

As shown in Figure 5, the scenario of total biodegradation of GNP/MWCNT/PLA nanowastes leads to a “very high” risk for release of GNPs and MWCNTs nanoparticles to the environment. This may pose questions associated with environmental and human risks due to local composting of post-consumer wastes, when those nanoparticles enter the soil. Combustion of nanowastes at heating temperatures of 500 °C may result in “unacceptable risk/hazard” from airborne GNPs and MWCNTs; however, by increasing the heating temperature to 850 °C, the risk decreases to “high”. Particularly, burning of nanowastes in open air (e.g., in landfills), which is a regular practice in underdeveloped regions, results in “high” risk. Therefore, such practices must be addressed and limited by the regulators, as it may affect more people and cause significant damage to the environment in those regions. The scenario of accident fire lead to “acceptable” risk, but it will have a local negative effect; therefore, preventive measures for safety have to be taken into account.

### 3.4. Safety Concerns

Bioplastic packaging is widely used these days for wrapping products from food to electronics to protect them from dust, bacteria and water vapor, and to maximize the lifetime of packaged products. Among the many different biopolymers used, poly(lactic acid) (PLA) possesses good mechanical property and cost-effectiveness necessary of biodegradable food packaging. However, PLA packaging suffers from poor water vapor and oxygen barrier properties compared to many petroleum-derived ones. A key challenge is, therefore, to simultaneously enhance both the water vapor and oxygen barrier properties by incorporation or encapsulation of graphene and carbon nanotubes into the PLA packaging [42,43]. Studies comparing the graphene nanoplates, GNPs and carbon nanotubes, shows CNTs are very rare, making it difficult to analyze their overall safety and risk [44,45]. In general, authors agree, that despite their common carbon structure, CNTs and GNPs are two very different nanomaterials, due to their different physical and chemical characteristics. Dimensions, surface chemistry and impurities are equally important for determining the aggregation, degradation and toxicological effects of CNTs and GNPs. Their shape (tubular vs. plane) and their dimensions (2D vs. 1D) are key structural differences. The CNTs tend to form entangled aggregates, and GNPs tend to stack in several layers. GNPs are characterized by a lower aspect ratio (length/width), greater surface area and better dispersion in most solvents, compared to CNTs. The colloidal dispersions of graphene can be obtained without metallic impurities, with high stability and less aggregation. All those characteristics could theoretically offer significant advantages of GNPs over CNTs, in terms of risk management and safety.

Our current study expands the upper safety concerns comparing GNPs and CNTs, by finding that large graphene nanosheets indeed release from PLA-based nanocomposite films at temperatures above the glass transition, during ultra-strong dynamic migration test. However, MWCNTs remain embedded in the polymer matrix if the PLA matrix is partly degraded. Moreover, GNPs and MWCNTs are found to remain unburned in the residue after combustion up to 650 °C, while at 850 °C only GNPs and carbon soot are found, but not MWCNTs.

In this research, we stress the safety concerns at the end-of-life of nanocomposite food packaging, related to different waste treatments, such as: biodegradation, combustion, burning in open air, and accident fire. Safety concerns may arise due to biodegradation and composting of nanowastes based on biodegradable polymers. Composting has the potential to transfer biodegradable plastics into useful soil amendment products by accelerated degradation. However, special care should be taken while handling local composting of biodegradable nanowastes to limit potential environmental risks due to the release of nanoparticles in the soil from the compost.

Combustion of nanocomposite wastes to produce energy has recently been discussed as a nanowastes management strategy, and thus the risks for nanoparticle emission during incineration of thermoplastic nanocomposites must be addressed and investigated. Our current study confirms that the combustion of PLA-based nanocomposites could form residue ashes containing unburned GNPs and MWCNTs; this is associated with “hazard” to “high risk”, depending on the temperature. The amount of unburned nanoparticles may be controlled by increasing the heating temperature above 500–850 °C; however, single GNPs and MWCNTs may also release in the combustion gas phase. Such release can be a source of risk in accidental scenarios, like fire, uncontrolled incineration/combustion, or the absence of nano-filtration of the combustion gas phase. Safety concerns arise about common practice in some regions for the burning of nanowastes in open air, e.g., in landfills or single-use disposable systems, due to the gradual increase of nanowastes from food packaging. As shown in this study, such regular practice leads to “high” risk for humans and the environment from airborne nanoparticles, such as GNPs and MWCNTs.

Based on the above study, we may propose a few safety measures for the prevention of risk from the packaging containing GNPs and MWCNTs at the end-life waste treatment: (i) Material safety data sheet (MSDS should contain clear information if the nanomaterial is safe for composting; (ii) specific labeling for prevention from composting should be adopted and printed on the packaging; (iii) regulatory limitations imposing the control on the combustion processes and exhaust gases will contribute to safety and risk prevention; (iv) regulatory measures imposing the limitation of burning of nanowastes in open air in landfills are required.

## 4. Conclusions

The release of graphene nanoplatelets and multiwall carbon nanotubes from polylactic-based film at the end-of-life of wastes treatment was investigated during degradation and combustion/burning. The released airborne nanoparticles and the degradation of the nanocomposite film during an ultra-strong dynamic migration test were confirmed by different visualization methods (TEM, AFM, SEM). Thermogravimetric analysis in air atmosphere was used to simulate the combustion of nanocomposite wastes. It was found that single graphene nanoplatelets of nanosized thickness and length of 100–1000 nm, as well as their micron-size loose aggregates, indeed release in relatively large amounts from the PLA nanocomposite film at high temperature dynamic treatment due to partial degradation of the PLA polymer. However, the release of the entangled MWSNTs is possible only after full degradation (hydrolysis) of the PLA matrix polymer.

Combustion or burning at 500 and 650 °C result in residue ash, which contains mainly single airborne GNPs and MWCNTs, while at 850 °C, the small amount of residue ash (~0.07%) contains only GNPs and amorphous carbon soot. Therefore, the MWCNTs fully degrade at the heating temperature of 850 °C, while the GNPs still remain in the residue.

New concerns with the end-of-life nanostructured materials emerged by adopting the 3-factors, C.E.L. model for risk assessment. The consequences (C) were determined from the amount of the released MWCNTs and GNPs, and were graded according to the recommended exposure limit of carbon nanotubes, REL = 1 μg/m^3^, proposed by NIOSH. The exposure (E) and likelihood (L) were estimated based on the E-classification method. Four scenarios are discussed: biodegradation by composting, combustion, burning in open air and accidental fire, which may lead to “very high”, “hazard” and “high” risk, respectively. Such treatment of nanowastes may pose a potential release of GNPs and MWCNTs into the environment, with all their associated environmental and human riskspresently not accounted for. Safety measures are proposed for the end-of-life phase of nanowastes in order to avoid or prevent the risks.

## Figures and Tables

**Table 1:** Thermal characteristics of PLA and GNP/MWCNT/PLA nanocomposite by TG analysis.

Sample	Temperature of Burning T_max_	T_onset_, °C	T_p_, °C	Weight Loss, % at T_max_	Residue Ash, %
Neat PLA	500 °C	219.8	356.5	99.63	0.30
Nanocomposite	500 °C	230.7	360.2	96.70	3.30
Nanocomposite	650 °C	230.1	361.3	98.50	1.05
Nanocomposite	850 °C	230.3	362.8	99.98	0.07

**Table 2:** The grades for Consequences (C), Exposure (E), Likelihood (L) and Risk (R) used in this study.

Consequences (C)	Exposure (E)	Likelihood (L)	Risk (R)
1 = minimal (≤100 REL)	0.5 = very rare (less than once a year)	0.2 = not imagine et al	<20 = minimal
3 = significant (100–1000 REL)	1 = rarely (once a year)	0.5 = almost impossible	20–70 = acceptable
7 = serious (1000–10,000 REL)	2 = sometimes (once a month)	1 = unbelievable, but long-term still possible	70–200 = high
15 = very serious (10,000–100,000 REL)	3 = happening (once a week)	3 = not be normal, but possible	200–400 = very high
40 = major damage (100,000–1,000,000 REL)	6 = regular (daily)	6 = completely possible	>400 unacceptable (hazard)
100 = crash (>1,000,000 REL)	10 = continuous	10 = almost certain	

**Table 3:** Risk assessment by CEL model for the release of GNPs, MWCNTs and CB from 3% GNP/MWCNT/PLA film during biodegradation, combustion, burning and accidental fire.

Scenario	Amount of Nano Wastes (kg)	Released Total Amount GNP/MWCNT/CB (kg)	Released GNP/MWCNT/CB Nanoparticles in 1 m^3^ Air (µg/m^3^)	Consequences (C) (× REL)	Exposure (E)	Likelihood (L)	Risk, (R) = C × E × L
Biodegradation	100	3	30,000	15	3	6	270 very high
Combustion 850–650–500 °C	100	0.07–1.05–3.3	70–10,500–33,000	1–15	10	10	100–1500 high-to-hazard
Burning of wastes at 500 °C	100	3.3	33,000	15	2	6	180 high
Accident fire 500 °C	100	3.3	33,000	15	0.5	3	22.5 acceptable
